# Tyrosylprotein Sulfotransferase-1 and Tyrosine Sulfation of Chemokine Receptor 4 Are Induced by Epstein-Barr Virus Encoded Latent Membrane Protein 1 and Associated with the Metastatic Potential of Human Nasopharyngeal Carcinoma

**DOI:** 10.1371/journal.pone.0056114

**Published:** 2013-03-05

**Authors:** Juan Xu, Xiyun Deng, Min Tang, Lili Li, Lanbo Xiao, Lifang Yang, Juanfang Zhong, Ann M. Bode, Zigang Dong, Yongguang Tao, Ya Cao

**Affiliations:** 1 Cancer Research Institute, Xiangya School of Medicine, Central South University, Changsha, Hunan, China; 2 Key Laboratory of Carcinogenesis and Cancer Invasion, Ministry of Education, Xiangya School of Medicine, Central South University, Changsha, Hunan, China; 3 Key Laboratory of Carcinogenesis, Ministry of Heath, Xiangya School of Medicine, Central South University, Changsha, Hunan, China; 4 Molecular Imaging Center, The First Affiliated Hospital of Xiangya School of Medicine, Central South University, Changsha, Hunan, China; 5 The Hormel Institute, University of Minnesota, Austin, Minnesota, United States of America; Ludwig-Maximilians University, Germany

## Abstract

The latent membrane protein 1 (LMP1), which is encoded by the Epstein-Barr virus (EBV), is an important oncogenic protein that is closely related to carcinogenesis and metastasis of nasopharyngeal carcinoma (NPC), a prevalent cancer in China. We previously reported that the expression of the functional chemokine receptor CXCR4 is associated with human NPC metastasis. In this study, we show that LMP1 induces tyrosine sulfation of CXCR4 through tyrosylprotein sulfotransferase-1 (TPST-1), an enzyme that is responsible for catalysis of tyrosine sulfation *in vivo*, which is likely to contribute to the highly metastatic character of NPC. LMP1 could induce tyrosine sulfation of CXCR4 and its associated cell motility and invasiveness in a NPC cell culture model. In contrast, the expression of *TPST-1* small interfering RNA reversed LMP1-induced tyrosine sulfation of CXCR4. LMP1 conveys signals through the epidermal growth factor receptor (EGFR) pathway, and EGFR-targeted siRNA inhibited the induction of TPST-1 by LMP1. We used a ChIP assay to show that EGFR could bind to the *TPST-1* promoter *in vivo* under the control of LMP1. A reporter gene assay indicated that the activity of the *TPST-1* promoter could be suppressed by deleting the binding site between EGFR and TPST-1. Finally, in human NPC tissues, the expression of TPST-1 and LMP1 was directly correlated and clinically, the expression of TPST-1 was associated with metastasis. These results suggest the up-regulation of TPST-1 and tyrosine sulfation of CXCR4 by LMP1 might be a potential mechanism contributing to NPC metastasis.

## Introduction

Nasopharyngeal carcinoma (NPC) is characterized by early metastatic spread, but the process of tumor cell dissemination is largely unknown [1]. A unique feature of NPC is its strong association with Epstein-Barr virus (EBV), which was the first human tumor virus identified as causally associated with various lymphoid and epithelial malignancies [2]. The EBV latent membrane proteins 1 and 2 (LMP1 and LMP2) are frequently expressed in NPC and have profound effects on cellular signaling networks and growth properties *in vitro* [3], [4]. In epithelial cells, LMP1 and LMP2 have been shown to affect differentiation, migration, anchorage independence, and tumorigenicity. A recent research report indicated that the combined expression of LMP1 and LMP2A promotes carcinoma development in a mouse carcinogenesis model [5]. Although most studies on LMP1 have focused on its primary oncogenic role in EBV-related malignancies, more recently LMP1 has also been implicated in their metastatic properties [6]. Activation of different signal transduction pathways mediates various downstream pathological effects of LMP1 expression, including cell proliferation, anti-apoptosis and metastasis [7], [8], [9]. In addition, LMP1 induces angiogenic factors such as the vascular endothelial growth factor (VEGF) through the induction of cyclooxygenase-2 (COX-2) [10], [11] and the JAK3/STAT signaling pathway [9], [12], thereby promoting invasion and metastasis of NPC cells.

The CXCR4 receptor and its chemokine ligand SDF-1α (CXCL12) are crucial for embryonic development, but have also been implicated in various pathologic conditions, including cancer metastasis [13], [14]. Cancer progression appears to be dependent on SDF-1α/CXCR4 signaling [15]. Increased CXCR4 expression in metastatic breast cancer cells causes these cells to seed secondary tumors by migrating into tissues and organs that express SDF-1α constitutively [16], [17]. Other types of cancer most likely exploit the same mechanism [17], [18]. Our previous study indicated that the expression of functional CXCR4 is associated with the metastatic potential of human NPC [19].

Accumulating evidence reveals that EBV is closely associated with expression of chemokines and their receptors, especially SDF-1/CXCR4. The EBV-encoded oncogenic protein LMP1 induces hypoxia inducible factor (HIF) expression [20], which can up-regulate CXCR4 and SDF-1 expression in NPC. Other reports showed that LMP1 regulates the expression of CXCR4, which is dependent on both IKKα and IKKβ in murine embryo fibroblasts (MEFs) [21]. LMP1 also down-regulates the expression of CXCR4 in B cells [22] and up-regulates the expression of CXCR4 in NPC C666-1 cells [23].

In recent years, tyrosine sulfation, as an important post-translational modification (PTM), has attracted much attention because of its mediation of chemokine receptor activity [24], [25], [26]. Evidence suggests that up to 1% of all tyrosine residues of the total protein content in an organism can be sulfated [27]. The function of CXCR4 was reportedly modified by sulfation at tyrosines 7, 12, and 21 in the CXCR4 N-terminal domain and Tyr21 of CXCR4 is considered as its main sulfation site [25], [28], [29]. The sulfate group at Tyr21 contributes substantially to the ability of CXCR4 to bind its ligand, SDF-1α. However, no evidence showing that sulfation of tyrosines in CXCR4 influences cell migration has yet been presented.

Tyrosylprotein sulfotransferases (TPSTs) are responsible for catalysis of tyrosine sulfation *in vivo.* Two different TPSTs (TPST-1 and TPST-2) have been identified [30], [31], and are broadly expressed in human and murine tissues and also co-expressed in the majority of cell types [32]. Peptides modeled on sulfation sites of the human C4α chain and heparin cofactor II are sulfated more efficiently by TPST-1 compared to TPST-2 [30]. A CXCR4 peptide can be modified at position 21 by expression of TPST-1[28], but the mechanisms of TPST-1 activation and function in cancer remain enigmatic.

We previously reported that the EBV-encoded LMP1 induced EGFR expression through the NF-κB signal transduction pathways, and increased the phosphorylation of EGFR in NPC cells [33]. After being phosphorylated, the new transcription factor EGFR was translocated into the nucleus to transactivate key regulators of the cell cycle, including cyclin D1 and cyclin E [34]. Also, emerging evidence suggests the existence of a direct EGFR-signaling pathway, which involves cellular transport of EGFR from the cell membrane to the nucleus, and transcriptional regulation of target genes such as *cox-2* [35]. Bioinformatic analysis (http://www.genomatix.de) revealed that the *TPST-1* gene (GenBank AF038009) contains EGFR binding sites, which are located in the 5′UTR region, i.e., TGTTT (located at -28-24). Therefore, we hypothesized that the EGFR might influence the tyrosine sulfation of CXCR4 by modulating TPST-1 to affect the binding of CXCR4 and its ligand. We concluded that LMP1 regulates the activity of CXCR4 through TPST-1 corresponding with the metastatic potential of NPC cells.

Based on this background information, we investigated whether the EBV oncogene *LMP1* could induce the sulfation of CXCR4 and its association with TPST-1 in this context. Further, we took advantage of EBV-LMP1 with its many known intracellular signaling pathways to explore the mechanistic link between EBV-LMP1 and TPST-1, and studied how the induction of TPST-1 by LMP1 might contribute to the highly metastatic character of NPC. We first show that LMP1 can activate the tyrosine sulfation of CXCR4 in metastasis in NPC cell culture models. We then demonstrated that in NPC cell culture models, up-regulation of TPST-1 depends on LMP1, and LMP1 can directly induce TPST-1 through the EGFR in nasopharyngeal epithelial cells. Finally, expression of TPST-1 correlates significantly with LMP1 protein expression as well as with metastasis in human NPC tissues.

## Materials and Methods

### Cell lines

Nasopharyngeal carcinoma cells, 5–8F and 6-10B [19], were kindly provided by Dr. H. M. Wang (Cancer Center, Sun Yat-sen University, P.R. China). HNE2-PSG5 is an EBV-LMP1-negative human NPC cell line constructed by transfecting a blank PSG5 vector into HNE2 cells. The HNE2-LMP1 cell line stably expresses LMP1 after the introduction of full-length cDNA into HNE2 cells [36]. The Tet-on-LMP1 HNE2 is a doxycycline-inducible NPC cell line in which the expression of LMP1 can be turned on by doxycycline (Dox, Sigma, St. Louis, MO) [37]. HNE2-PSG5, HNE2-LMP1, 5-8F and 6-10B cells were grown in RPMI 1640 (GIBCO, Grand Island, NY) supplemented with 10% fetal bovine serum (FBS), 1% glutamine, and 1% antibiotics. Tet-on-LMP1 HNE2 cells were cultured in RPMI 1640 medium with 10% FBS, 100 mg/L G418 and 50 mg/L of hygromycin. The human embryonic kidney cell line, HEK293 (ATCC CRL-1573) was obtained from the cell bank of the Xiangya School of Medicine (Changsha, China) and was cultured in DMEM (GIBCO) supplemented with 10% FBS, 1% glutamine, and 1% antibiotics. All cell lines were cultured at 37°C in a humidified incubator containing 5% CO_2_. Cells in logarithmic growth phase were used in all experiments.

### Plasmid constructs and small interfering RNA

*pEGFP-CXCR4* (WT-CXCR4) was constructed by cloning the whole *CXCR4* (GeneBank accession no. NM_003467.2) coding fragment into the *KpnI/XhoI* sites of the *pEGFP-N1* vector. *pEGFP-mut-CXCR4* (MUT-CXCR4) was point mutated by altering Tyr21 in the N-terminal of CXCR4 to phenylalanine (Phe) and then inserted into the *pEGFP-N1* vector. The *pSG5*-based expression vector for wildtype LMP1 derived from the B95.8 EBV strain was kindly provided by Dr. Izumi (Brigham and Women's Hospital, Boston, MA). The *pcDNA4-TPST1* expression plasmid was constructed by cloning the whole *TPST1* (GeneBank accession no. AF038009) coding fragment into the *KpnI/XhoI* sites of the *pcDNA4/HisMax© Vector A.* The *TPST-1* siRNA (sc-41075) and *EGFR* siRNA (sc-29031) were purchased from Santa Cruz Biotechnologies (Santa Cruz, CA).

The *TPST1* promoter is a 704 bp fragment containing the human EGFR binding site (GXP_1827191, www.genomatix.de) and was obtained by amplification from human HNE2 cellular genomic DNA. The sense primer (5′- *ggtacc*gtatatactgtatatactgagt-3′) used in this reaction carried the *Kpn I* cloning site, whereas the antisense primer (5′-*ctcgag*ccggtgatggca ttccatgg-3′) contained the *Xho I* site. The italicized nucleotides represent restriction endonuclease recognition sites. This fragment was inserted into the *KpnI/XhoI* sites of the *pGL3-Basic* vector (Promega, Madison, WI) and the plasmid was designated as *pluc-TPST1-EGFR*. The EGFR motif mutants (designated as *pluc-TPST1mtEGFR*) from *pluc-TPST1-EGFR* were generated by PCR based on an overlap extension technique. The primers used for generating mutations were: 5′-ctgaagtagac**tgtcc**atggcc-3′ and

5′-gttattatag**agata**ttttg-3′. PCR-amplified fragments carrying the desired mutations were then cloned into the *Kpn I/Xho I* sites of the *pGL3-Basic* vector. The expected mutations and the integrity of the enhancer were confirmed by automated sequencing using an Applied Biosystems sequencer and software (Foster City, CA).

### Preparation of cell lysates and Western blot analysis

Cells were harvested and washed twice with ice-cold phosphate-buffered saline (PBS), and then disrupted in lysis buffer (10 mM Tris–HCl pH 8.0, 1 mM EDTA, 2% SDS, 5 mM dithiothreitol, 10 mM phenylmethyl sulfonylfluoride, 1 mM Na_3_VO_4_, 1 mM NaF, 10% (vol/vol) glycerol, protease inhibitor cocktail tablet) for 30 min on ice and centrifuged at 15,000×g for 10 min. The supernatant fraction was collected as a whole cell lysate. Protein concentration was determined using the BCA Assay Reagent (Pierce, Rockford, IL). Total proteins (100 μg) from various cell preparations and rainbow molecular weight markers (Amersham Pharmacia Biotech, Amersham, UK) were separated by 8% SDS-polyacrylamide gel electrophoresis and then electrotransferred onto nitrocellulose membranes. The membranes were blocked for 2 h with buffer containing 5% non-fat milk in PBS with 0.05% Tween-20, and then incubated with different primary antibodies overnight at 4°C. After the second wash, the membranes were incubated with an anti-rabbit (sc-2004, Santa Cruz), anti-mouse (sc-2005, Santa Cruz) or anti-goat (sc-2020, Santa Cruz) horseradish peroxidase-conjugated secondary antibody for 1 h at room temperature. Blots were developed with an enhanced chemiluminescence detection kit (ECL; Pierce). The following antibodies were used for Western blotting: mouse LMP1 monoclonal antibody (M0897, DAKO, Copenhagen, DK), rabbit anti-human CXCR4 (AB1846, Millipore, Jaffrey, NH), anti-sulfotyrosine (05-1100, Millipore, Danver, MA), anti-TPST1 (sc-25033, Santa Cruz, CA), anti-EGFR (sc-03-G, Santa Cruz), anti-β-actin (sc-8432, Santa Cruz).

### Real time RT-PCR

The expression levels of TPST-1 were determined by SYBR green real-time reverse transcription-PCR (RT-PCR). Total RNA from different human nasopharyngeal cells was extracted using Trizol reagent (Invitrogen, Carlsbad, CA). Quantitative determination of RNA levels was performed in triplicate in three independent experiments. Real-time PCR and data collection were performed with an ABI 7500 sequence detection system. The housekeeping gene *β-actin* was used as an internal control to normalize the expression levels of different genes. For human *TPST-1*, the forward primer, 5′-acccacctaactacggaa-3′ and the reverse primer, 5′-ctgaaaaggcagcaatct-3′ yield a 172-bp product. For human *β-actin*, the forward primer, 5′- ttccagccttccttcctggg -3′, and the reverse primer, 5′- ttgcgctcaggaggagcaat -3′, yield a 224-bp product.

### Flow cytometry

Nasopharyngeal carcinoma cells were grown to subconfluency, detached with cold Dulbecco's PBS (5 mM EDTA), and washed with fluorescence-activated cell sorting buffer (5 mM EDTA, 0.1% NaN_3_, and 1% FCS, in Dulbecco's PBS). After incubation with a mouse LMP1 monoclonal antibody (M0897, DAKO) for 30 min on ice, the cells were stained with an FITC-labeled secondary antibody and examined for LMP1 expression by flow cytometry (BD, San Diego, CA).

### Labeling and immunoprecipitation

Cells were labeled with [^35^S] cysteine and [^35^S] methionine or [^35^S] sulfate for 1 day. Cysteine and methionine labeling media contained 50 mCi each of [^35^S] cysteine and [^35^S] methionine per milliliter of Dulbecco's modified Eagle's medium lacking both amino acids (D0422, Sigma). Sulfate labeling media contained 125 mCi [^35^S] sulfate/ml sulfate-free media (F12-11765, GIBCO). Labeled cells were disrupted in 1% *N*-dodecyl-*β*-D-maltoside (Calbiochem, Gibbstown, NJ) in PBS containing protease inhibitor mixtures for mammalian cells (Roche, Basel, CH). After cellular debris was removed by centrifugation at 14,000 rpm for 10 min at 4°C, the supernatant fraction was used for immunoprecipitation with the CXCR4 antibody. Immunoprecipitates were washed twice with 1% *N*-dodecyl-*β*-D-maltoside/PBS containing 0.5% SDS and once with PBS. Reducing Laemmli sample buffer was added and samples were treated at 50°C for 10–15 min before analysis by SDS-PAGE. A 4-fold greater volume of [^35^S] sulfate-labeled samples were analyzed relative to samples radiolabeled with cysteine and methionine.

### Chemotaxis

Chemotaxis assays were performed using 48-well chemotaxis chambers (Neuro Probe, Gaithersburg, MD) as described previously [19]. Aliquots of 27- to 29-AL assay medium (RPMI 1640 containing 1% bovine serum albumin and 30 mM HEPES) with different concentrations of SDF-1α (300-28A, PeproTech, Rocky Hill, NJ) were placed in the lower wells of the chamber. The cell suspension (50 ml, 1×10^6^ cells/ml) was placed in the upper wells. The upper and lower wells were separated by a polycarbonate membrane (Neuro Probe, 10-mm pore size), which was pre-coated with 50 mg/ml collagen type I (Collaborative Biomedical Products, Bedford, MA). After incubation at 37°C for 5 h, the filter was removed, stained, and the cells that migrated across the filter were counted under a light microscope after coding the samples. The results are expressed as chemotaxis index, which represents the fold increase in the number of migrated cells in response to chemoattractants over the spontaneous cell migration in response to control medium.

### Transwell invasion Assay

The transwell invasion assay was performed according to Costar's Transwell procedure. Briefly, cells were seeded onto ECM pre-coated gel porous upper chamber inserts (1×10^5^ each well) and allowed to invade for 36 h. After 36 h, the inserts were inverted and stained with 1% crystal violet. The numbers of invaded cells were observed and counted using a light microscope. Five fields were randomly chosen and the number of penetrated cells was counted.

### Chromatin immunoprecipitation (ChIP) assay

Cells were grown to 70–80% confluence in 10-cm plates, and were then processed for ChIP assays using a kit (EZ-ChIP, Millipore, Billerica, MA). DNA was sheared to a size of 200-1,000 base pairs (bp) prior to performing the immunoprecipitation (IP). The sheared chromatin was pre-cleared by incubating with protein G beads for 1 h at 4°C to reduce non-specific background. Pre-cleared chromatin was then incubated with anti-EGFR (sc-03-G, Santa Cruz), anti-rabbit IgG (2 mg, sc-2005, Santa Cruz), or no antibody overnight at 4°C. The kit's negative control IgG and positive control anti-RNA Polymerase II were also used in parallel reactions.

Protein G bead incubation, washing of ChIP reactions, DNA elution from protein G, cross-link reversal, RNA removal and purification of eluted DNA were performed following the protocol included in the kit. Isolated DNA was subjected to RT-PCR and analyzed by agarose gels stained with ethidium bromide. Q-PCR was used to detect the binding of TPST-1 with EGFR. The primers for the *TPST-1* promoter domain, which included the EGFR binding region, that were used in the ChIP assays were as follows: 5′-tgtccatggcctgaacatt-3' and 5'- gcttcagctttccaaccatc-3' (94 bp).

### Transient transfection and luciferase assay

Cells were seeded in 24-well plates before transfection. The *pluc-TPST1-EGFR* or *pluc-TPST1mEGFR* plasmid was co-transfected with an internal control, *pRL-SV40*, using the Lipofectamine 2000 transfection reagent (Invitrogen) following the manufacturer's instructions. Cells were harvested at 24 h after transfection and lysates were analyzed for luciferase activity using the Dual Luciferase Reporter assay (Promega) according to the manufacturer's directions. The *luciferase reporter* plasmids were co-transfected with *pRL-SV40* to correct for variations in transfection efficiency. The relative luciferase activity was normalized to the value of *pRL-SV40* activity. The data are represented as means ± S.D. of 3 independent experiments performed in triplicate.

### Immunohistochemical analysis

Nasopharyngeal carcinoma tissues from routine diagnostic biopsy specimens were obtained from the Department of Pathology, Tumor Hospital of Hunan Province, Changsha, China for immunohistochemical analysis. We obtained written permission from each patient to use his/her biopsy for the current research study. Immunohistochemistry was performed as previously described [19]. Primary antibodies to detect LMP1 and TPST-1 were the same as used for Western blotting. Previously identified LMP1-positive NPCs served as positive controls for LMP1 [6] and TPST-1 was first observed by immunohistochemistry. NPC specimens were evaluated independently by two authors (Juan Xu and Xiyun Deng) with knowledge of the clinical data and then reviewed by a pathologist (Liang Zeng). Two examiners each selected two representative fields of >200 tumor cells and counted both the stained cells and the total number of tumor cells. The average percentage of stained cells was used to calculate the LMP1 and TPST-1 expression scores. Staining was repeated at least twice in sequential sections to assess reproducibility.

### Statistical analysis

All statistical calculations were performed with the statistical software program SPSS12.0. The Pearson's correlation coefficient was used to analyze correlations between the expression of LMP1and TPST-1 in NPC. The expression of LMP1and TPST-1 in relation to clinical data was analyzed with the x^2^ test. Significant differences between various groups were determined with the Student's t test.

## Results

### LMP1 up-regulates the level of tyrosine sulfation of CXCR4 in NPC cells

In our previous study, we found that functional CXCR4 is expressed in highly metastatic NPC 5-8F cells, but not in nonmetastatic NPC 6-10B cells [19]. Moreover, LMP1 expression has been shown to be higher in 5–8F cells compared to 6–10B cells [38]. This implies that LMP1 could influence invasion and metastasis of NPC through the tyrosine sulfation of CXCR4 (S-CXCR4), which is the functional form of CXCR4.

**Figure 1:**
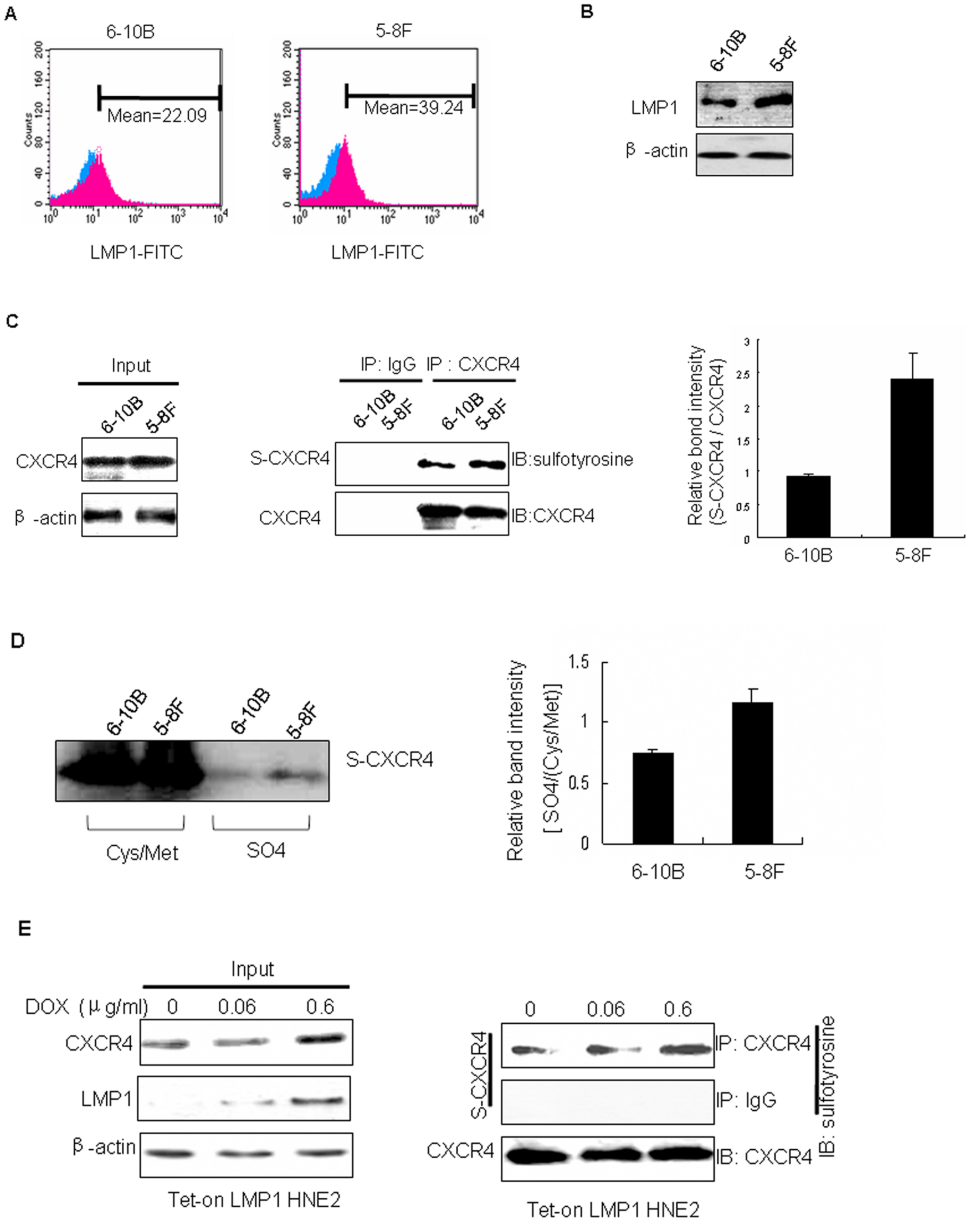
LMP1 up-regulates the level of tyrosine sulfation of CXCR4 in NPC cells. The expression of LMP1 (red) was detected in 5–8F and 6–10B cells by flow cytometry. Control immunoglobulin staining is shown (blue). Data are shown as the mean fluorescence intensity. (B) LMP1 expression was assessed in 5–8F and 6–10B whole cell lysates by Western blot. β-Actin was used as a control to verify equal protein loading. (C) Tyrosine sulfation of CXCR4 (S-CXCR4) expression was assessed in 6–10B and 5–8F cells by immunoprecipitation and Western blot (IP-Western blot). CXCR4 expression was assessed in 6–10B and 5–8F whole cell lysates by Western blot, β-Actin was used as a control to verify equal protein loading. CXCR4 was used as a control to verify equal protein loading and IgG was used as a negative control for S-CXCR4 in IP-Western blot. The expression level of S-CXCR4 was estimated by densitometry and presented as a ratio to the loading control CXCR4. The data are shown as means ± S.D. of three independent experiments performed in triplicate. (D) S-CXCR4 expression was assessed in 6–10B and 5–8F cells by labeling and immunoprecipitation. The sulfation level of Cys/Met was used as a control to verify equal protein labeling. The labeling and immunoprecipitation were performed as described in “Materials and methods.” The expression level of S-CXCR4 was estimated by densitometry and presented as a ratio to the labeling control Cys/Met. The data are shown as means ± S.D. of three independent experiments performed in triplicate. (D) S-CXCR4 expression was assessed in 6–10B and 5–8F cells by (E) The effect of inducible LMP1 on the induction of tyrosine sulfation of CXCR4 was assessed using the Tet-on-LMP1-HNE2 cell line. Tet-on-LMP1-HNE2 cells were stimulated with the indicated doses of Dox for 24 h. Whole cell lysates were used to measure expression of S-CXCR4 by IP-Western blot and LMP1 by Western blot. CXCR4 was used as the loading control for S-CXCR4. β-Actin was used as the loading control for LMP1 and IgG was used as a negative control for S-CXCR4.

We first confirmed by flow cytometry and Western blot that LMP1 expression is greater in highly metastatic 5–8F cells compared to nonmetastatic 6–10B cells (Fig. 1A–B). We observed tyrosine sulfation of CXCR4 in these two cell lines using labeling and immunoprecipitation (IP) or IP and Western blot analysis. Results (Fig. 1 C–D) indicate that the level of tyrosine sulfation of CXCR4 is higher in 5–8F cells compared to 6-10B cells. Further, we found that LMP1 increased the tyrosine sulfation of CXCR4 in a dose-dependent manner (Fig. 1E) in the tetracycline-regulated LMP1-expressing NPC cell line. These results indicate that LMP1 can up-regulate tyrosine sulfation of CXCR4 in NPC cells.

### LMP1 up-regulates cellular chemotactic activity and invasiveness through the tyrosine sulfation of CXCR4 in NPC

**Figure 2:**
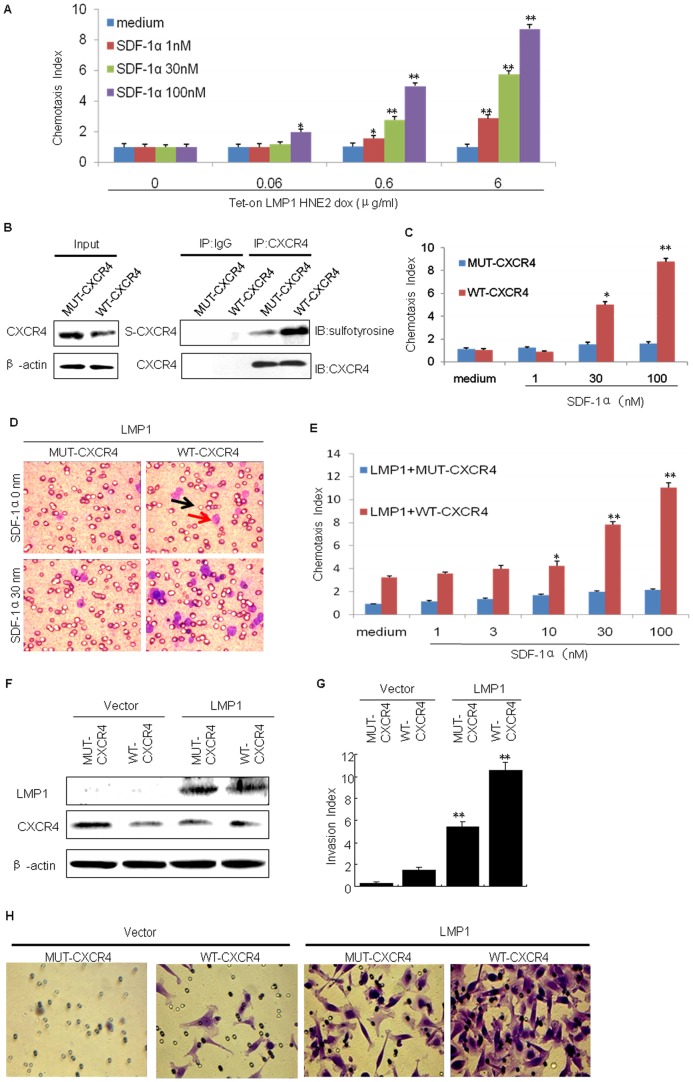
LMP1 up-regulates cellular chemotactic activity and invasiveness through the tyrosine sulfation of CXCR4. (A) Tet-on LMP1 HNE2 cells were incubated for 24 h with medium containing the indicated concentration(s) of Dox. The migration of Tet-on-LMP1 HNE2 cells in response to SDF-1α was measured using chemotaxis chambers. Data are shown as tumor dose-response curves of Tet-on-LMP1 HNE2 cells migrating toward Dox. The asterisks indicate a statistically significant difference (**, *p*<0.01; *, *p*<0.05). (B) HEK293 cells were transiently transfected with plasmids to express *WT-CXCR4* or *MUT-CXCR4*. At 24 h post-transfection, cells were analyzed by Western blot for CXCR4 expression and by IP for expression of S-CXCR4. β- Actin and CXCR4 were used as control to verify equal protein loading. IgG was used as a negative control for S-CXCR4. (C) Chemotactic activity of SDF-1α in HEK293 cells. Data are shown as dose-response curves of HEK293 cells migrating toward SDF-1α. The asterisks indicate a statistically significant difference (* *, *p*<0.01, *, *p*<0.05). (D) The migration of HEK293 cells in response to SDF-1α was measured using chemotaxis chambers. Representative photographs show tumor cells migrating across polycarbonate filters. The migration of HEK293 cells transfected with *WT-CXCR4* and *LMP1* or *MUT-CXCR4* and *LMP1* plasmids in response to medium alone or 30 nmol/L SDF-1α, respectively, is shown. The black arrow indicates one of the micropores in the filter and the red arrow indicates a migrated cell. (E) Data are shown as dose-response curves of HEK293 cells migrating toward SDF-1α. The asterisks indicate a statistically significant difference (**, *p*<0.01; *, *p*<0.05). (F) HNE2-PSG5 and HNE2-LMP1 cells were transiently transfected with plasmids to express *WT-CXCR4* or *MUT-CXCR4*. At 24 h post-transfection, cells were analyzed by Western blot for CXCR4 and LMP1 expression, β-Actin was used as control to verify equal protein loading. (G) The effect of the *WT-CXCR4* or *MUT-CXCR4* on migration of nasopharyngeal carcinoma cells was assessed. HNE2-LMP1 cells were transiently transfected with *WT-CXCR4* or *MUT-CXCR4* plasmids and migration was measured using the Matrigel invasion assay. The asterisks indicate a statistically significant difference (**, *p*<0.01; *, *p*<0.05). (H) Effects of *WT-CXCR4* or *MUT-CXCR4* on invasion and metastasis of cells. Tumor cells migrating across the Matrigel were photographed. The data are representative of three experiments with similar results.

Post-translational modifications of the amino termini of chemokine receptors, and, in particular, tyrosine sulfation, play a critical role in the ability of these receptors to associate with their natural ligands [26], [39]. We confirmed that LMP1 up-regulates tyrosine sulfation of CXCR4, and the functional CXCR4 induces cellular chemotactic activity and metastasis in NPC [19]. We now determined whether LMP1 could regulate cellular chemotactic activity and invasiveness through the tyrosine sulfation of CXCR4. We treated Tet-on LMP1 HNE2 cells for 24 h with various doses of Dox and SDF-1α. Results of these chemotactic experiments indicated that cells migrated in response to the CXCR4 ligand, SDF-1α, in a concentration-dependent manner and also in an LMP1 dose-dependent manner (Fig. 2A). To further confirm these findings in non-tumor cells, we transiently transfected WT-CXCR4 or MUT-CXCR4 into HEK293 cells, which are negative for expression of LMP1 and CXCR4. Only HEK293 cells transfected with WT-CXCR4 migrated in response to the CXCR4 ligand SDF-1α in a concentration-dependent manner (Fig. 2B–C). LMP1 increased the cellular migration induced by WT-CXCR4 (Fig. 2D-E). In contrast, HEK293 cells transfected with MUT-CXCR4 did not show any migratory response to SDF-1α (Fig. 2B–C). Moreover, LMP1 did not increase the migration of MUT-CXCR4 cells (Fig. 2D–E). These results further confirmed that LMP1 enhances the functional activity of CXCR4 by up-regulating tyrosine sulfation of CXCR4.

Next, we studied cellular invasiveness with the use of a Matrigel invasion chamber system. Results indicated that HNE2-LMP1 cells transfected with the WT-CXCR4 plasmid were more invasive than HNE2-LMP1 cells transfected with the MUT-CXCR4 plasmid (Fig. 2F–H), suggesting that LMP1 up-regulates the invasiveness of HNE2-LMP1 cells by tyrosine sulfation of CXCR4. Thus, LMP1 induces cellular motility and invasion of NPC cells by stimulating tyrosine sulfation of CXCR4.

### TPST-1 is required for tyrosine sulfation of CXCR4 by LMP1 in NPC

**Figure 3:**
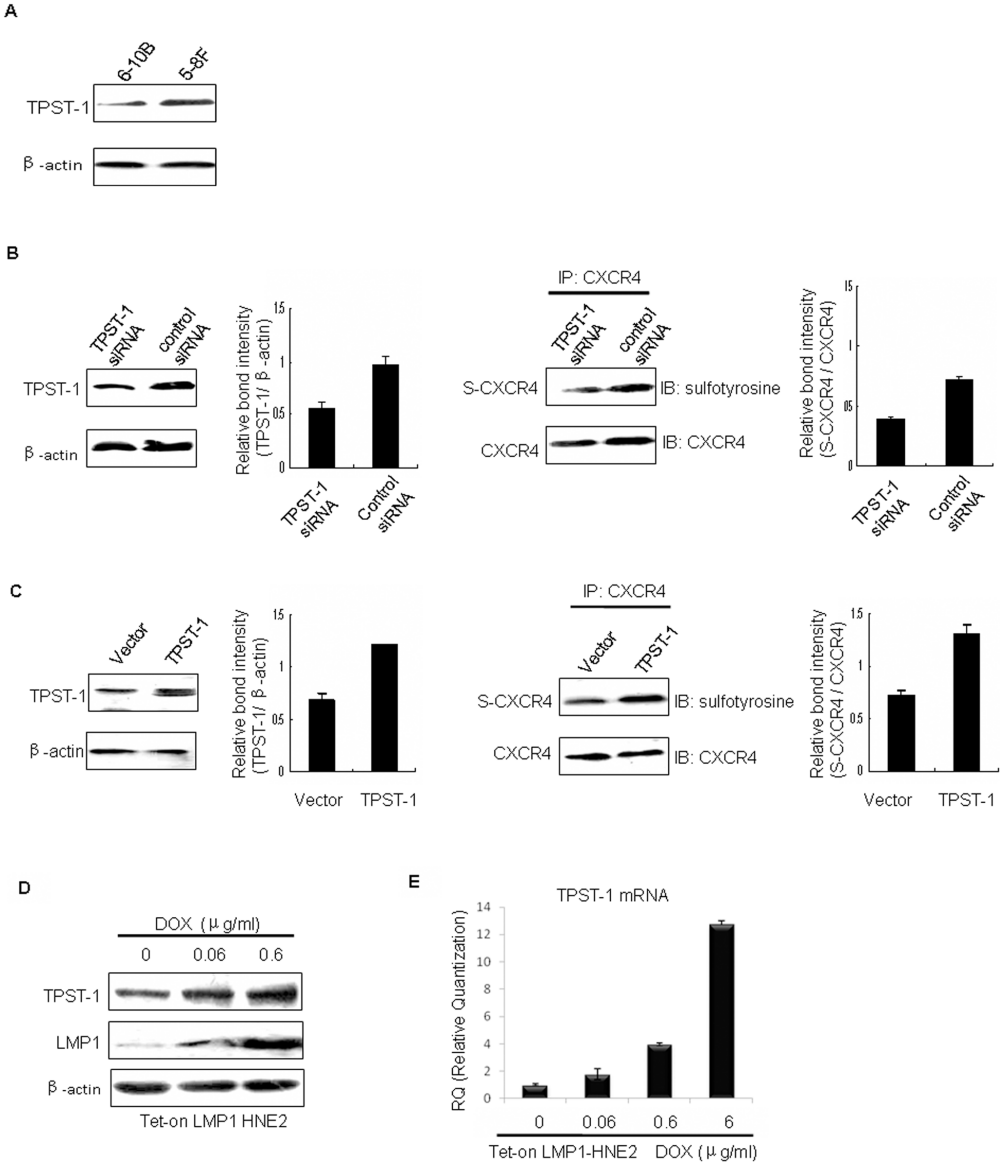
LMP1 up-regulates tyrosine sulfation of CXCR4 through TPST-1. (A) xpression of TPST-1 in 5–8F and 6–10B cells. TPST-1 expression was assessed in 5–8F and 6–10B whole cell lysates by Western blot and β-actin was used as a control to verify equal protein loading. (B) TPST-1siRNA reduces tyrosine sulfation of CXCR4 in 5–8F cells. 5–8F cells were incubated for 24 h with medium containing 50 pM *TPST-1* siRNA and TPST-1 expression was measured in whole cell lysates by Western blot. β-Actin was used as a loading control for TPST-1and CXCR4 was used as a loading control for S-CXCR4.The expression level of TPST-1 and S-CXCR4 was estimated by densitometry and presented as a ratio to the loading control. The data are shown as means ± S.D. of three independent experiments performed in triplicate. (D) S-CXCR4 expression was assessed in 6–10B and 5–8F cells by (C) TPST-1 induces tyrosine sulfation of CXCR4 in 6–10B cells. 6–10B cells were transfected with TPST-1 expression plasmids for 24 h. TPST-1 expression was assessed in whole cell lysates by Western blot. β-Actin was used as a control for equal loading of TPST-1 and CXCR4 was the control for S-CXCR4. The expression level of TPST-1 and S-CXCR4 was estimated by densitometry and presented as a ratio to the loading control. The data are shown as means ± S.D. of three independent experiments performed in triplicate. (D) S-CXCR4 expression was assessed in 6-10B and 5–8F cells by (D) Tet-on-LMP1 HNE2 cells were stimulated with the indicated doses of Dox for 24 h. Whole cell lysates were used for Western blot to detect expression of LMP1 and TPST-1. β-Actin was used as a control to verify equal protein loading. (E) Tet-on-LMP1-HNE2 cells were incubated for 24 h with medium containing the indicated concentration(s) of Dox. Total RNA was isolated from cells and subjected to real-time RT-PCR, using “primer 5” software designed to amplify *TPST-1* and *actin* mRNAs. The software used delta-delta Ct for measuring mRNA levels of TPST-1. b-b-Actin was used as a control to verify equal loading.

TPSTs are known to be very important for tyrosine sulfation of substrate proteins [31], [40]. Therefore, we determined whether the ability of LMP1 to induce tyrosine sulfation of CXCR4 is attributable to TPST-1. First, we found that TPST-1 protein expression is higher in 5–8F cells compared to 6–10B cells (Fig. 3A). Further, we examined the significance of the higher TPST-1 expression by transducing TPST-1 siRNA into 5–8F cells exhibiting high LMP1 expression and transducing TPST-1 expression plasmids into 6–10B cells that exhibit low LMP1 expression. We then investigated whether changes in TPST-1 expression could lead to any changes in tyrosine sulfation of CXCR4. Results indicate that reducing TPST-1 expression in 5–8F cells, which express high levels of LMP1, reduced the level of tyrosine sulfation of CXCR4 (Fig. 3B). Increasing TPST-1 expression in 6-10B cells, which express low levels LMP1, up-regulated the level of tyrosine sulfation of CXCR4 (Fig. 3C). Collectively, these observations indicate that TPST-1 is required for LMP1-induced tyrosine sulfation of CXCR4.

To examine whether LMP1 is responsible for induction of TPST-1 expression, we used an LMP1-inducible expression NPC cell line, Tet-on-LMP1 HNE2. The expression of LMP1 in Tet-on-LMP1 HNE2 cells is tightly regulated by doxycycline modulation [37]. We observed that when LMP1 expression was simulated by the addition of various doses (0, 0.06, 0.6 μg/ml) of Dox, Dox induced a marked increase of LMP1 in a dose-dependent manner (Fig. 3D). Following the addition of various concentrations of Dox, TPST-1 in Tet-on-LMP1 HNE2 cells was also substantially elevated in an LMP1 dose-dependent manner (Fig. 3D). In order to determine if the observed increase in TPST-1 protein expression was related to an effect of LMP1 on *TPST-1* mRNA levels, Tet-on-LMP1 HNE2 cells were treated with Dox and the levels of *TPST-1* mRNA were monitored by Q-PCR. Treatment with Dox induced a marked dose-dependent increase in *TPST-1* mRNA (Fig. 3E). The results indicate that LMP1 is capable of inducing expression of the TPST-1 protein and mRNA in NPC cells. These findings indicate that LMP1 could increase the production of TPST-1 by NPC cells at both the mRNA and protein levels.

### LMP1 induces expression of TPST-1 through the EGFR pathway in NPC

**Figure 4:**
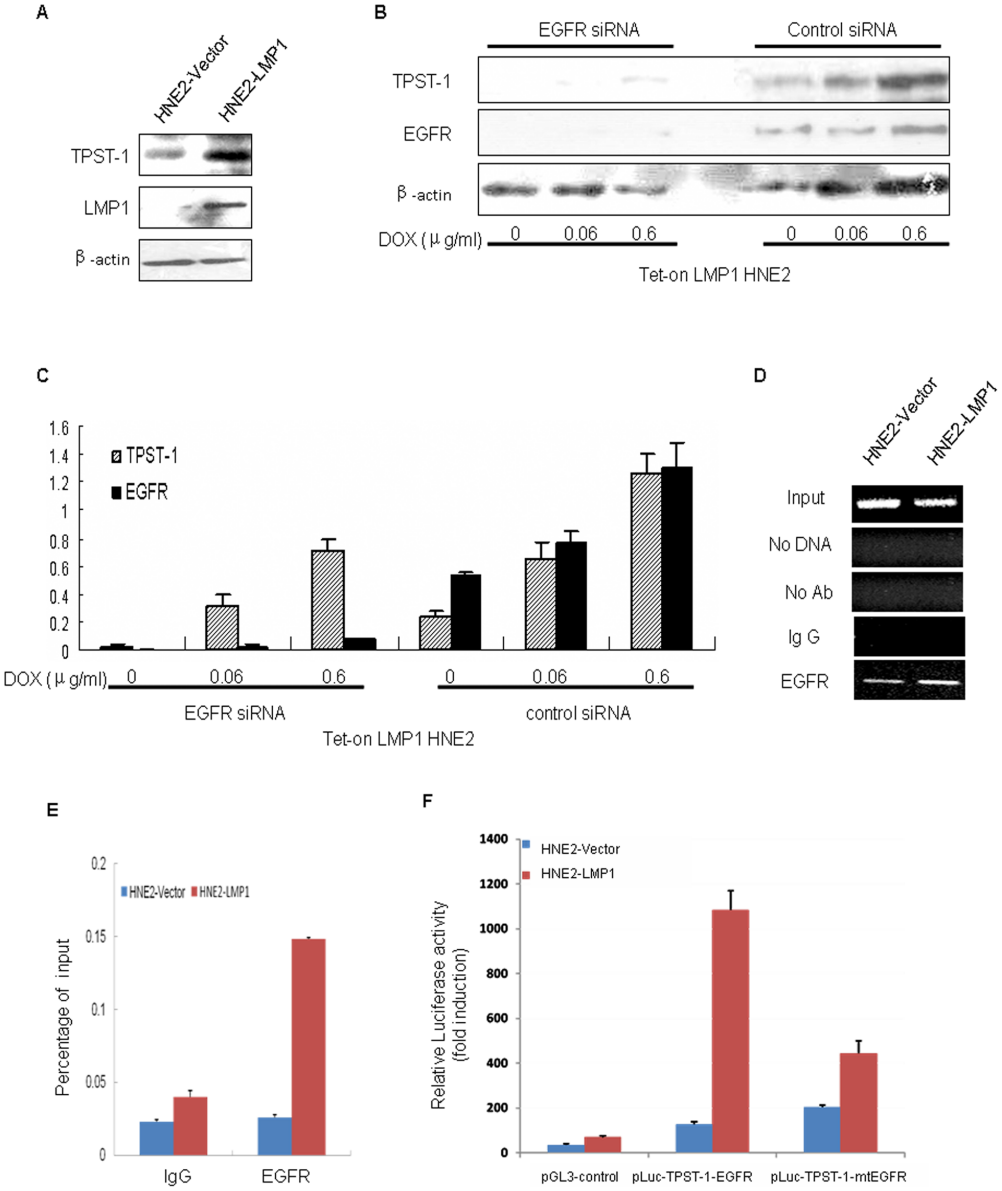
LMP1 induces the expression of TPST-1 by activating EGFR. (A) LMP1 and TPST-1 expression was assessed in HNE2-PSG5 and HNE2-LMP1 whole cell lysates by Western blot. β-Actin was used as a control to verify equal protein loading. (B) *EGFRsiRNA* decreases expression of TPST-1 in Tet-on-LMP1 HNE2 cells. Tet-on-LMP1 HNE2 cells were stimulated with the indicated doses of Dox for 24 h followed by incubation with 50 pM *EGFRsiRNA* for an additional 24 h. EGFR and TPST-1 expression was measured in whole cell lysates by Western blot. β-Actin was used as a control to verify equal protein loading. (C) Expression level of each protein was estimated by densitometry and presented as a ratio to the loading control β-actin. The data are shown as means ± S.D. of three independent experiments performed in triplicate. (D) S-CXCR4 expression was assessed in 6–10B and 5–8F cells by (D) ChIP-PCR analysis of EGFR binding site on the promoter of *TPST-1* in HNE2-PSG5 and HNE2-LMP1 cells. The cross-linked chromatin that was precipitated with specific antibodies as indicated. The input fraction represents the positive control. Negative controls include a sample with no chromatin, a sample with no antibody, and a sample with a nonspecific antibody (IgG). The precipitated DNA was analyzed by PCR using primers that amplified a 94-bp region, which included the EGFR site in HNE2-PSG5 and HNE2-LMP1 cells. (E) LMP1 promotes the binding of the EGFR and the *TPST-1* promoter. ChIP-Q-PCR analysis shows that LMP1 promotes EGFR binding to the *TPST-1* promoter. Anti-EGFR was used to identify the EGFR binding sites on the *TPST-1* promoter in HNE2-PSG5 and HNE2-LMP1cells. The binding activity of each protein is given as percentage of total input. (F) LMP1 augments *TPST-1* promoter activity through the EGFR. The constructs carry either wild-type sequences or mutations (depicted by crosses) in the EGFR sites are shown. Transient transfection and luciferase reporter assays were performed as described in “Materials and methods” to compare the transcriptional activation of the *TPST-1* promoter in nasopharyngeal carcinoma cells. The relative luciferase activity is normalized to the value of *Renilla* luciferase activity. Results are expressed as fold induction of the activity of vector-transfected HNE2-PSG5 cells, which was assigned a value of 1. The data are shown as means ± S.D. of the 3 independent experiments performed in triplicate.

Our preliminary work confirmed that LMP1 increased the expression of the transcription factor EGFR. From genetic information, we found that EGFR binding sites are present in the *TPST-1* promoter domain. Therefore, we concluded that LMP1 might mediate TPST-1 by activating EGFR signal transduction. We observed that TPST-1 expression in HNE2-LMP1 cells was higher than that in HNE2-PSG5 cells (Fig. 4A). After introducing *EGFR* siRNA into Tet-on LMP1 HNE2 cells for 24 h to block EGFR expression, we found that TPST-1 expression was decreased (Fig. 4B–C). Further, ChIP assay data showed that EGFR could bind to the *TPST-1* promoter *ex vivo* under the control of LMP1 (Fig. 4D–E). Reporter gene analysis showed that LMP1 increased *TPST-1* promoter activity; and the activity of the *TPST-1* promoter was decreased by mutating the binding site of EGFR and TPST-1 (Fig. 4 F). These results indicated that LMP1 induced TPST-1 expression in an EGFR-dependent manner in NPC cells.

### Expression of TPST-1 is associated with LMP1 expression in NPC

**Figure 5:**
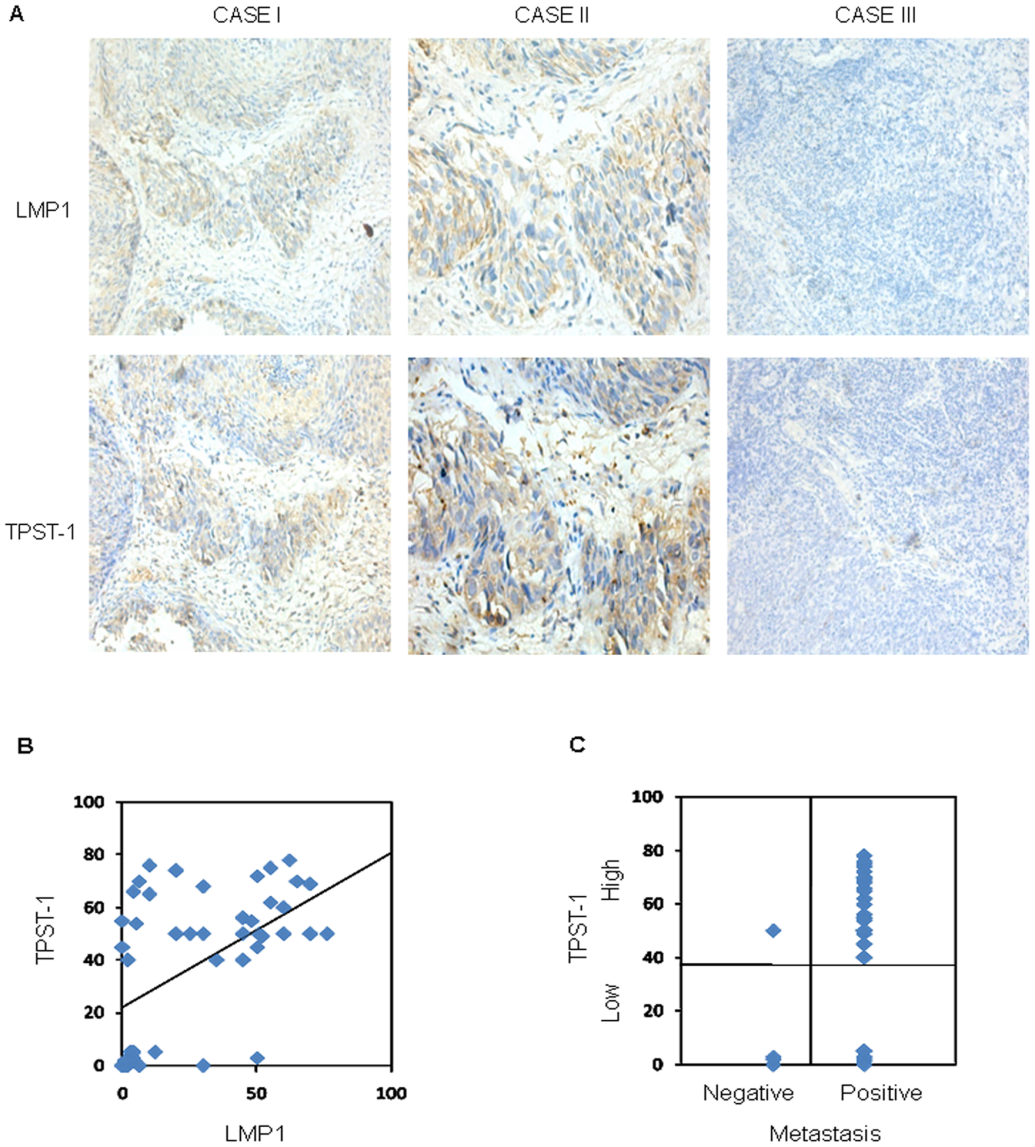
Expression of LMP1 and TPST-1 in NPC tissues as detected by immunohistochemistry. (A) The LMP1 protein is visible at the cell membrane and in the cytoplasm of nests of tumor cells. The TPST-1 protein is localized mainly in the cytoplasm of tumor cells. Case I (original magnification, 200X) and Case II (original magnification, 400X) are representative cases showing high LMP1 and TPST-1 expression. Case III (original magnification, 200X) is a representative case showing no expression of LMP1 or TPST-1. (B) Expression scores for LMP1 and TPST-1 in all 46 NPC specimens. Average percentages of tumor cells staining for LMP1 and TPST-1 are plotted (see “Materials and methods”). Expression scores for LMP1 and TPST-1 were significantly correlated, Pearson correlation coefficient, r = 0.514, *p*<0.01; *p* = 0.0001. (C) Expression of TPST-1 is significantly associated with metastasis in NPC tissues. *p*<0.01; *p* = 0.007.

The studies in cell culture models suggested that induction of TPST-1 expression by LMP1 contributes to tumor cell invasion and metastasis. Therefore, we determined whether a correlation exists between expression of TPST-1 and LMP1 in NPC. TPST-1and LMP1 expression was analyzed by immunohistochemistry in 46 human NPC tissues. The TPST-1 protein was detected mainly in the cytoplasm and the LMP1 protein was detected in both the membranes and cytoplasm of the tumor cells (Fig. 5A). Case I and Case II are a representative of results in which both LMP1 and TPST-1 are highly expressed. In contrast, Case III is representative of results when LMP1 and TPST-1 are not expressed. The distribution of all 46 cases based on LMP1 and TPST-1 protein levels is plotted in Fig. 5B. The expression of TPST-1 showed a linear dependence on the expression of LMP1 (r = 0.514; *p*<0.0001; Fig. 5B). These results combined with those obtained from the cell culture systems suggest that LMP1 is a major inducing signal for TPST-1 in NPC.

### TPST-1 levels correlate directly with human tumor malignant progress

The question to be addressed is whether TPST-1 is associated with the malignant progress of NPC, especially metastasis, which is the most frequent finding in NPC [41]. We therefore analyzed the relationship of the expression of TPST-1 with metastasis in NPC. The results indicate that the TPST-1 protein was detected in tumor cells in 1of 6 nonmetastatic human NPC tissues and in 29 of 40 lymph node metastases (Fig. 5C). Furthermore, the expression of TPST-1 in metastasis-positive cases is significantly higher than that in metastasis-negative cases (*p*<0.05, *p* = 0.007) (Fig. 5C). In addition, we also found that TPST-1 expression is associated with the clinical stage of NPC tissues, in that the expression of TPST-1 in clinical stage III-IV cases is significantly higher than in clinical stage I-II (*p*<0.05) (Table 1).

**Table 1:** The expression of LMP1 and TPST1 in NPC tissues.

Clinic stage	LMP1			TPST-1		
	+	-	χ^2^	+	-	χ^2^
I-II	1	5	5.660*	1	5	7.170*
III-IV	27	13		29	11	

* Significantly increased rate of LMP1-positive and TPST1-positive specimens in stage III-IV than in stage I-II nasopharyngeal carcinoma as evaluated with χ^2^ test (*P*<0.05).

## Discussion

Although the levels of CXCR4 expression correlate directly and strikingly with the degree of metastasis in NPC [19] and in several human carcinomas [13], [42], [43], [44], the functional role of tyrosine sulfation of CXCR4 in these tumors is largely unknown. In our previous study, both metastatic and nonmetastatic nasopharyngeal carcinoma cell lines express CXCR4 at the mRNA and protein levels, but functional CXCR4 is only found in cell lines with metastatic properties [19]. Whether a human tumor viral oncogene could regulate tyrosine sulfation of CXCR4 has not yet been examined. The major findings reported here are that tyrosine sulfation of CXCR4 is up-regulated by TPST-1 and associated with the principal EBV oncogene *LMP1* in one of the most invasive EBV-associated malignancies, NPC. Indeed, NPC stands out among head and neck tumors for its invasive and metastatic propensity [41]. Moreover, the viral oncoprotein *LMP1* can clearly induce tyrosine sulfation of CXCR4, through TPST-1, and inhibition of TPST-1 by *TPST-1* siRNA can reverse the sulfation of CXCR4. The significance of these results is emphasized by the direct correlation between the level of TPST-1 and metastatic potential of NPC.

We previously reported that the functional expression of CXCR4 induced metastasis in human NPC. Here, we focus on NPC and the association of the EBV oncogene *LMP1* with tyrosine sulfation of CXCR4, which is the functional form of CXCR4. LMP1 could induce tyrosine sulfation of CXCR4, which is associated with cell motility and invasiveness *in vitro*. This report is the first to show the induction of tyrosine sulfation of CXCR4 by a viral oncogene and clarifies the vital role of TPST-1. By using an online microarray database (http://www.oncomine.com), we found that TPST-1 is overexpressed in breast cacinoma [45], oral squamous cell carcinoma [46], and Barretina Sarcoma [47] compared to expression in normal tissues. TPST-1 is also involved in invasion and metastasis of head and neck carcinoma [48]. These results not only are important in explaining the metastatic character of EBV-related malignancies such as NPC, but also provide new insight for the pathogenesis of tyrosine sulfation of CXCR4 in human carcinomas.

In our study, LMP1 up-regulated both TPST-1 mRNA and protein levels in a dose-dependent manner in NPC cells. The association between expression of TPST-1 and LMP1 in NPC tissues further supports the existence of such an induction mechanism. However, the mechanism explaining how TPST-1 can be regulated by LMP1 in cancer progression is a key question yet to be answered and remains quite obscure. Our preliminary studies showed that LMP1 is involved with several signal transduction pathways, including the EGFR, which triggers the activation of several different target genes to affect the biological behavior of tumor cells [33], [34], [49], [50]. EGFR is involved in invasion and metastasis of several human carcinomas, and correlates directly with CXCR4 expression [51], [52]. Reports indicate that activation of EGFR can regulate activity of sulfatases in renal cell carcinoma metastasis to promote sulfation of glycosphingolipids and enhance tumor cell invasion and metastasis [53]. Results of our current study clearly provide one answer to this question by demonstrating the induction of TPST-1 by the EBV oncogene *LMP1* mediated through the EGFR, and provides insights into how TPST-1 can be activated. However, other transcription factors are also able to play a certain part in LMP1-induced up-regulation of TPST-1 in NPC by adjusting the *TPST-1* promoter activity. Thus, further study is under way to more fully address the issue of TPST-1 regulation in NPC. Nevertheless, a potential role for TPST-1 in mediating the metastasis of NPC through sulfation of CXCR4 shown in our study suggests that TPST-1 should be considered as a molecular target for small molecule directed therapy.
